# The Agreement Between Transthoracic and Transesophageal Echocardiography in the Assessment of Right Ventricular Diastolic Dysfunction Grades in Adult Patients Undergoing Cardiac Surgery: A Prospective Observational Study

**DOI:** 10.7759/cureus.70976

**Published:** 2024-10-07

**Authors:** Nehal C Singh, Indranil Biswas, Bhupesh Kumar, Krishna Prasad Gaurav, Sanjeev Naganur, Pankaj Aggarwal

**Affiliations:** 1 Anaesthesia and Intensive Care, Postgraduate Institute of Medical Education and Research, Chandigarh, Chandigarh, IND; 2 Cardiology, Postgraduate Institute of Medical Education and Research, Chandigarh, Chandigarh, IND; 3 Cardiothoracic and Vascular Surgery, Postgraduate Institute of Medical Education and Research, Chandigarh, Chandigarh, IND

**Keywords:** agreement, diastolic dysfunction, intraoperative transesophageal echocardiography, right ventricle (rv), transthoracic echocardiography (tte)

## Abstract

Introduction

The importance of right ventricular (RV) diastolic function in cardiac surgery cannot be overstated, as it significantly affects prognosis and long-term outcomes. Conventionally, RV diastolic dysfunction (RVDD) is assessed and graded using criteria from either the American Society of Echocardiography (ASE) or the British Society of Echocardiography (BSE), with measurements done by transthoracic echocardiography (TTE). However, during cardiac surgery, perioperative echocardiographic evaluation is done predominantly by transesophageal echocardiography (TEE). This study aimed to assess the agreement between TTE and TEE in grading RVDD using both ASE and BSE criteria.

Methods

Key two-dimensional (2D) and Doppler parameters were measured in 81 patients undergoing cardiac surgery by both TTE and TEE after anesthesia induction within 10 minutes of each other, under similar hemodynamic, anesthetic, and ventilatory conditions. RVDD gradings were done separately by TTE and TEE with both ASE and BSE criteria using the measured values of the key parameters by TTE and TEE, respectively. RVDD gradings derived from TTE were compared with those derived from TEE. The tricuspid inflow Doppler and tricuspid annular tissue Doppler parameters were measured in TEE in both mid-esophageal RV inflow-outflow (MERVIO) and deep transgastric RV inflow-outflow (DTGRVIO) views. Gradings were done separately for both views of TEE by using the Doppler values measured in the respective views (TEE-MERVIO and TEE-DTGRVIO). The TTE-derived RVDD grades were compared with those derived by both TEE-MERVIO and TEE-DTGRVIO. Weighted κ values were used to assess observed agreement beyond chance. Inter-rater reliability of the RVDD grades derived by both TTE and TEE (both views) was also checked. Individual 2D and Doppler parameters were compared between TTE and TEE in terms of Bland-Altman limits of agreement.

Results

As per ASE criteria, disagreement of RVDD by ≥1 grade was seen in 43 (53.1%) patients and by 2 grades in eight (9%) patients when comparing TTE and TEE-MERVIO, yielding a weighted κ of 0.14 (p=0.123). Disagreement by ≥1 grade was observed in 32 (39.5%) patients and by 2 grades in 10 (12.3%) patients when comparing TTE and TEE-DTGRVIO, yielding a weighted κ of 0.3 (p=0.002). Using the BSE Criteria, disagreement of RVDD grades occurred in nine (11.1%) patients when comparing TTE and TEE-MERVIO, yielding an unweighted κ of 0.25 (p=0.295). Disagreement occurred in 12 (14.8%) patients when comparing TTE and TEE-DTGRVIO, yielding an unweighted κ of 0.260 (p=0.187). There was almost perfect agreement between independent raters regarding both TTE- and TEE-derived RVDD grades per the ASE criteria, and substantial to almost perfect agreement per BSE criteria. Bland-Altman analysis of paired data between the TTE- and TEE-measured values of individual 2D and Doppler parameters showed wide limits of agreement.

Conclusions

This study revealed, at best, only fair agreement between TTE and TEE in grading RVDD. The measured 2D and Doppler echocardiographic parameters showed wide limits of agreement between TTE and TEE. We recommend further research to develop a TEE-based algorithm for grading RVDD, and to evaluate the prognostic effectiveness of perioperative TEE for predicting adverse clinical outcomes associated with RVDD.

## Introduction

Right ventricular (RV) diastolic function is an important parameter to be assessed in the context of cardiac surgery, as it aids in the prognosis of cardiac surgery and helps assess long-term outcomes [1]. Perioperative echocardiography is a vital tool to assess RV diastolic function. In the setting of cardiac surgery, hemodynamic imbalances with RV dysfunction and stroke volume variation are not uncommon. In such instances, administration of fluids would be futile as a vicious cycle sets in, where RV dysfunction subsequently affects cardiac output, thus failing to correct the hemodynamic instability [2,3]. Therefore, it is critical to assess and eventually manage RV dysfunction in the perioperative period. The diastolic function of the right heart is assessed using various parameters studied through transthoracic echocardiography (TTE). These validated parameters include Doppler velocities of trans tricuspid flow, deceleration time, tissue Doppler velocities of the tricuspid annulus and isovolumetric relaxation time, right atrial (RA) area, hepatic venous Doppler, and inferior vena cava (IVC) dimension and collapsibility.

Both the American Society of Echocardiography (ASE) and the British Society of Echocardiography (BSE) have published guidelines to diagnose as well as grade RV diastolic dysfunction (RVDD) [4,5]. Intraoperative echocardiography, on the other hand, is predominantly done using transesophageal echocardiography (TEE). Studies comparing RV systolic function between TTE and TEE have found only fair agreement when performed under similar hemodynamic conditions [6]. Poor agreement has been found between TTE- and TEE-measured left ventricular (LV) diastolic dysfunction grades [7]. However, there is scarce literature comparing TTE and TEE for RVDD grading. Therefore, in the present study, we aimed to compare RVDD grades assessed by TTE-measured and TEE-measured parameters.

## Materials and methods

Hypothesis

TEE-guided measurement of the right ventricular dysfunction grades has poor agreement with the diastolic dysfunction grades assessed through TTE.

Objectives

The primary objective of the study was to find out the agreement between TTE and TEE in the measurement of RVDD grades. The secondary objective of the study was to find out whether the TEE view has the best agreement with TTE for the assessment of RVDD grades.

Study design, duration, and protocol

This prospective, observational study was conducted in the cardiac surgical unit of our institute from July 15, 2023, to June 30, 2024. The study commenced after receiving clearance from the Institutional Ethics Committee (IEC INT/2023/DM-1133) and subsequent registration with the Clinical Trial Registry of India (CTRI/2023/07/054689). Informed consent was taken from the patients to enroll them in the study.

Inclusion and exclusion criteria

Patients in the age group of 20-60 years of either gender undergoing coronary artery bypass grafting (CABG) or valve repair/replacement procedures were included in the study. Patients were excluded if they met any of the following criteria: contraindications to insertion of a TEE transducer; weighing less than 25 kg; organic disease of the tricuspid valve (TV); tricuspid ring/prosthesis; more than mild tricuspid regurgitation, or tricuspid annular calcification; pacemaker; heart rhythm other than normal sinus rhythm; poor TTE window; pericardial disease; congenital shunt lesions; and requirement of inotropic/vasopressor support prior to induction of anesthesia.

Anesthesia and echocardiography protocol

After the patients entered the operating room (OR), five-lead electrocardiogram, pulse oximetry, non-invasive blood pressure, and BiSpectral Index (BISTM) monitors were attached. An invasive arterial catheter and a central venous catheter were inserted following the infiltration of 2% lignocaine at the respective insertion sites. General anesthesia was induced with fentanyl, propofol, and vecuronium by using the balanced anesthesia technique. The induction method was chosen by the anesthesia consultant present in the OR. Anesthesia was maintained using inhalational isoflurane in an O_2_/air mixture with a target BISTM of 50. Both the TTE and TEE examinations were performed after induction of general anesthesia, endotracheal intubation, and initiation of controlled mechanical ventilation.

All the echocardiographic examinations were performed using a GE Vivid E9 (GE Healthcare Vingmed Ultrasound AS, Horten, Norway) workstation. After the induction of general anesthesia and insertion of the endotracheal tube, a 6VT-D TEE probe was inserted using standard maneuvers. TTE was performed by an anesthesia/cardiology fellow/faculty with more than three years of experience in Focus-Assessed Transthoracic Echocardiography (FATE)/TTE with the patient in the supine position, using an M5S-D (1.5-4.5 MHz) transthoracic probe. Thus, RV-focused apical four-chamber views were obtained. TV diastolic inflow velocities were obtained with pulsed-wave (PW) Doppler by placing a sample volume of 3 mm at the tip of the TV leaflets after aligning the Doppler beam parallel to the RV inflow. Tricuspid annular tissue Doppler velocities were acquired with tissue Doppler imaging (TDI) by placing a PW Doppler sample volume of 5 mm at the lateral edge of the tricuspid annulus after aligning the Doppler beam parallel to the movement of the lateral tricuspid annulus.

The right atrial area (RAA) was measured from the apical four-chamber view during the end of ventricular systole on the frame just before the TV opening. IVC diameter was measured in the subcostal long-axis view perpendicular to the IVC long axis, 1-2 cm from the RA junction at end-expiration. Loop for measuring RV wall thickness was obtained in the subcostal four-chamber view with a zoom over the RV inferior wall. Hepatic venous Doppler was obtained in the subcostal long-axis view with PW Doppler by placing a sample volume of 3 mm in the hepatic vein, 1-2 cm inside from its drainage point into the IVC. Continuous-wave (CW) Doppler trace of the pulmonary artery was obtained in parasternal short-axis view at the level of the aortic valve with the cursor aligned along the main pulmonary artery.

After completing the TTE examination, the anesthesia/cardiology faculty/fellow left the OR, and subsequently, a faculty/fellow, other than the faculty/fellow posted in the operating room and having more than three years of experience in performing TEE, entered the operating room and performed the TEE examination before surgical incision. The maximum time allowed between the TTE and the TEE acquisitions was 10 minutes, during which the anesthetic depth (monitored objectively by BISTM) and mechanical ventilation settings were kept constant. If heart rate, mean arterial pressure, or central venous pressure deviated by more than 10% of those while performing TTE, or the patient required any inotropes/vasopressors before obtaining all the required TEE images, the patient was excluded from the study.

TV diastolic inflow velocities were obtained from mid-esophageal right ventricular inflow-outflow (MERVIO) view and deep transgastric right ventricular inflow-outflow (DTGRVIO) views with PW Doppler by placing a sample volume of 3 mm at the level of the tip of the TV leaflets after aligning the Doppler beam parallel to the RV inflow. Tricuspid annular tissue velocities were also obtained in the MERVIO and DTGRVIO views with TDI by placing a PW sample volume of 5 mm at the tricuspid annulus after aligning the Doppler beam parallel to the movement of the tricuspid annulus. RA area was obtained in a modified mid-esophageal four-chamber view by rotating the TEE probe clockwise from the mid-esophageal four-chamber view to show the RA maximally.

IVC size and hepatic vein Doppler were obtained in lower esophageal IVC and hepatic vein views by inserting the TEE probe into the lower esophageal position near the gastroesophageal junction and increasing the multiplane angle to 60 degrees. Hepatic venous Doppler was obtained with PW Doppler by placing a sample volume of 3 mm in the hepatic vein, 1-2 cm inside from its drainage point into the IVC. IVC diameter was measured perpendicular to the IVC long axis, 1-2 cm from the RA junction at end-expiration. CW Doppler trace of the pulmonary artery (PA) was obtained in the mid-esophageal ascending aortic short-axis view with the cursor aligned along the main pulmonary artery. Loop for measuring RV wall thickness was obtained in the DTGRVIO view using zoom mode over the RV inferior wall.

Doppler and tissue Doppler images were obtained at end-expiration apnea with a sweep speed of 66.67 mm/sec, with a single image containing waveforms of at least five cardiac cycles. All TTE and TEE two-dimensional (2D) video loops, PW Doppler, and tissue Doppler images were stored, and measurements and grading were done offline using Echopac software (version 113) by a single evaluator (EV1) with more than five years of experience. For each individual parameter, the average value of five cardiac cycles was taken as the final value.

RVDD grade was determined for individual patients separately by following the criteria recommended by the ASE [4] and the BSE [5]. Values of various parameters obtained by TTE for each individual subject were used to determine RVDD grade by TTE. In each subject, values of RA area, RV wall thickness, IVC diameter, hepatic venous Doppler, PA CW trace by TEE, together with the values of the tricuspid inflow Doppler and tricuspid annular tissue Doppler parameters obtained in the MERVIO view, were used to determine the RVDD grade by TEE (MERVIO). Similarly, values of RA area, RV wall thickness, IVC diameter, hepatic venous Doppler, PA CW trace by TEE, together with the values of the tricuspid inflow Doppler and tricuspid annular tissue Doppler parameters obtained in the DTGRVIO view, were used to determine the RVDD grade by TEE (DTGRVIO).

Interobserver variability of the parameters measured by TTE and TEE was tested through a review and analysis of the saved images and loops, and by another independent, experienced (>5 years of experience in both TTE/FATE and TEE) evaluator (EV2). Intraobserver variability was tested by repeat analysis one month after the initial analysis by the initial evaluator (EV1), using the same saved images and loops. The independent evaluator (EV2) also graded the diastolic dysfunction using the parameters analyzed by him/her by both ASE and BSE criteria.

Sample size calculation

Based on an expected agreement (weighted Cohen’s κ) of 0.3 with a precision of 0.15, a confidence level of 80%, and an expected drop-out rate of 10%, a sample size of 80 was required for this study.

Statistical analysis

The agreement between the RVDD grades determined by TTE and TEE measurements was analyzed by creating contingency tables and calculating the proportion of weighted observed agreement, the proportion of weighted expected agreement, and finally the linear weighted (Cicchetti Allison) Cohen’s κ coefficient. Strength of agreement was classified according to values of κ, such that values in the ranges of <0.20, 0.21-0.40, 0.41-0.60, 0.61-0.80, and >0.80 were considered poor, fair, moderate, good/substantial, and very good/almost perfect agreement, respectively. The reliability of the echocardiographic measurements was evaluated using the intraclass correlation coefficient (ICC) from a two-way mixed-effects model with absolute agreement. An ICC of >0.80 indicated good reliability.

Agreement between EV1 and EV2 regarding RVDD grading was analyzed by creating contingency tables and calculating the proportion of weighted observed agreement, the proportion of weighted expected agreement, and finally the weighted (Cicchetti Allison) Cohen’s κ coefficient. Normal distribution of the hemodynamic, ventilatory, and anesthetic depth parameters was checked by the Kolmogorov-Smirnov test and visual Q-Q plotting. Student’s t-test (for data distributed normally) or Mann-Whitney test (for data not having normal distribution) were used to compare the hemodynamic, ventilatory, and anesthetic depth parameters between TTE and TEE time points. A p-value <0.05 indicated a statistically significant difference. All statistical analyses were performed using SPSS Statistics V22.0 (IBM Corp., Armonk, NY) software and the DATAtab (DATAtab e.U. Graz, Austria) online statistics calculator.

## Results

**Figure 1:**
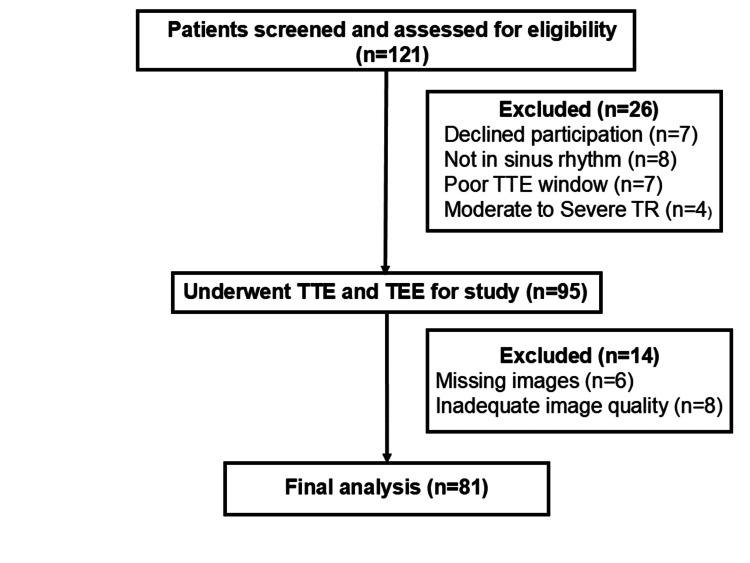
Chart showing patient flow from screening to analysis TEE: transesophageal echocardiography; TR: tricuspid regurgitation; TTE: transthoracic echocardiography

A total of 121 patients were approached for the study, of which seven declined to participate, and 19 were excluded for meeting the exclusion criteria set for the study. Among the remaining 95 patients, 14 were excluded because they had echo images that were either missing or of poor quality. Ultimately, 81 patients, were selected for the final analysis (Figure 1).

The mean (standard deviation) age of the participants was 49 (13) years, and 56 (69.1%) of them were male; 34 (41%) of the patients were hypertensive, and 15 (18.5%) were diabetic. Thirty-seven (45.7%) underwent isolated CABG surgery, whereas 24 (29.6%) and 10 (12.3%) underwent isolated aortic valve and mitral valve procedures, respectively (Tables 1-2). The hemodynamic, anesthetic, and ventilatory parameters were comparable between TTE and TEE time points (Table 3).

**Table 1:** Demographic parameters of the study population BMI: body mass index; BSA: body surface area; SD: standard deviation

Variable	Values
Age, years, mean ±SD	48.6 ±13
Male/female, n/n	56/25
Weight, kg, mean ±SD	63.32 ±11.3
Height, m, mean ±SD	1.63 ±0.09
BMI, kg/m^2^, mean ±SD	23.66 ±3.63
BSA, m^2^,mean ±SD	1.68 ±0.19

**Table 2:** Operative characteristics of study population (N=81) AVR: aortic valve replacement; CABG: coronary artery bypass graft; DVR: double valve replacement; MVR: mitral valve replacement

Sl. no.	Surgical procedure	Number of patients (percentage)
1	CABG	37 (45.7%)
2	AVR/OZAKI	24 (29.6%)
3	MVR	10 (12.3%)
4	Aortic procedure	4 (4.9%)
5	CABG + valve procedure	4 (4.9%)
6	DVR	2 (2.4%)

**Table 3:** Comparison of hemodynamic and ventilatory parameters during TTE and TEE P<0.05 is considered statistically significant (Mann-Whitney U test) BIS: BiSpectral Index; CVP: central venous pressure; EtCO_2_: end-tidal carbon dioxide; HR: heart rate; MAP: mean arterial pressure; SpO_2_: pulse oximeter saturation; PEEP: positive end-expiratory pressure; P mean: mean airway pressure; P peak: peak airway pressure; TEE: transesophageal echocardiography; TTE: transthoracic echocardiography

Variable	TTE (n=81)	TEE (n=81)	P-value
	Mean ±SD	Mean ±SD	
HR	70 ±13	70 ±12	0.849
MAP	78 ±9	78 ±8	0.936
CVP	6 ±4	6 ±3	0.983
	Median (range)	Median (range)	
SpO_2_	99 (96–100)	99 (96–100)	0.413
EtCO_2_	34 (27–40)	34 (27–39)	0.842
BIS	49 (45–52)	50 (44–52)	0.164
P peak	13 (8–22)	13 (8–21)	0.527
PEEP	5 (5–5)	5 (5–5)	0.998
P mean	7 (4–11)	7 (4–11)	0.829

According to TTE-derived parameters, 49 (60.5%), 11 (13.6%), and 21 (25.9%) patients were found to have normal, impaired relaxation, and pseudo-normal RV diastolic filling patterns, respectively, as per the ASE criteria (Table 4); whereas 75 (92.6%) and six (7.4%) patients were found to have normal and abnormal RV diastolic function, respectively, based on the BSE criteria (Table 5). According to parameters measured by TEE (MERVIO), 37 (45.7%), 10 (12.3%), and 34 (42%) patients were found to have normal, impaired relaxation, and pseudo-normal RV diastolic filling patterns, respectively, based on the ASE criteria (Table 4); whereas 69 (85.2%) and 12 (14.8%) patients were found to have normal and abnormal filling patterns, respectively, based on the BSE criteria (Table 5).

**Table 4:** RVDD grades measured by TTE and TEE according to ASE criteria ASE: American Society of Echocardiography; RVDD: right ventricular diastolic dysfunction; TEE-DTGRVIO: transesophageal echocardiographic deep transgatric right ventricular inflow-outflow view; TEE-MERVIO: transesophageal echocardiographic mid-esophageal right ventricular inflow-outflow view; TTE: transthoracic echocardiography

View	RVDD grade (n=81), n (%)		
	Normal	Impaired relaxation	Pseudo-normal
TTE	49 (60.5%)	11 (13.6%)	21 (25.9%)
TEE-MERVIO	37 (45.7%)	10 (12.3%)	34 (42%)
TEE-DTGRVIO	51 (63%)	15 (18.5%)	15 (18.5%)

**Table 5:** RVDD Grades measured by TTE and TEE according to BSE criteria BSE: British Society of Echocardiography; RVDD: right ventricular diastolic dysfunction; TEE-DTGRVIO: transesophageal echocardiographic deep transgatric right ventricular inflow-outflow view; TEE-MERVIO: transesophageal echocardiographic mid-esophageal right ventricular inflow-outflow view; TTE: transthoracic echocardiography

View	RVDD grade (n=81), n (%)	
	Normal	Abnormal
TTE	75 (92.6%)	6 (7.4%)
TEE-MERVIO	69 (85.2%)	12 (14.8%)
TEE-DTGRVIO	74 (91.4%)	7 (8.6%)

According to parameters measured by TEE (DTGRVIO), 51 (63%), 15 (18.5%), and 15(18.5%) patients were found to have normal, impaired relaxation, and pseudo-normal filling patterns, respectively, based on the ASE criteria (Table 4); whereas 74 (91.4%) and seven (8.6%) patients were found to have normal and abnormal filling patterns, respectively, based on the BSE criteria (Table 5). None of the patients were found to have a “restrictive” filling pattern based on either ASE or BSE criteria, using parameters measured by TTE, TEE (MERVIO), and TEE (DTGRVIO). As a “restrictive” filling pattern was not found in any of the patients, 3x3 contingency tables were prepared, and linear weighted (Cicchetti Allison) kappa statistics were used for comparing RVDD grading between TTE and TEE by ASE criteria, and 2x2 contingency tables were prepared and unweighted kappa statistics was used for comparison using BSE criteria.

RVDD by TTE vs. TEE (MERVIO) using ASE criteria

Out of 81 paired measurements, disagreement by ≥1 RVDD grade was seen in 43 (53.1%) patients and by 2 grades in eight patients (9%), among which five patients had a deterioration of RVDD by 2 grades from TTE to TEE (MERVIO), and three patients had improvement of RVDD by 2 grades (Table 6). The observed weighted agreement proportion was 0.68, whereas the expected weighted agreement proportion was 0.63, yielding a weighted κ of 0.14 (95% CI: −0.05 to 0.34; p=0.123), indicating poor agreement, which likely occurred due to random chance.

**Table 6:** Contingency table assessing agreement between RVDD grading measured by TTE and TEE using the ASE criteria (tricuspid inflow Doppler and tricuspid annular tissue Doppler values measured in the MERVIO view) Disagreement of ≥1 grade: 53.1%. Disagreement of 2 grades: 9.9%. Observed weighted agreement proportion: 0.68. Expected weighted agreement proportion: 0.63. Linear (Cicchetti Allison) weighted κ=0.14 (95% CI: −0.05 to 0.34; p=0.123), used to evaluate the observed level of agreement beyond chance ASE: American Society of Echocardiography; CI: confidence interval; RVDD: right ventricular diastolic dysfunction; TEE-MERVIO: transesophageal echocardiographic mid-esophageal right ventricular inflow-outflow view; TTE: transthoracic echocardiography

RVDD grades	RVDD grade (ASE criteria) by TEE (MERVIO), n (%)				
RVDD grade (ASE criteria) by TTE		Normal	Impaired relaxation	Pseudo-normal	Total
	Normal	25 (30.9%)	19 (23.5%)	5 (6.2%)	49
	Impaired relaxation	9 (11.1%)	10 (12.3%)	2 (2.5%)	21
	Pseudo-normal	3 (3.7%)	5 (6.2%)	3 (3.7%)	11
	Total	37	34	10	81

RVDD by TTE vs. TEE (DTGRVIO) using ASE criteria

Out of 81 paired measurements, disagreement by ≥1 RVDD grade was observed in 32 patients (39.5%) and by 2 grades in 10 patients (12.3%), of which six patients had deterioration of RVDD by 2 grades from TTE to TEE (DTGRVIO), and four patients had improvement of RVDD by 2 grades (Table 7). The observed weighted agreement proportion was 0.74, whereas the expected weighted agreement proportion was 0.63, yielding a weighted κ of 0.3 (95% CI: 0.1 to 0.51; p=0.002), indicating statistically significant fair agreement that was unlikely to have occurred due to random chance.

**Table 7:** Contingency table assessing agreement between RVDD grading measured by TTE and TEE using the ASE criteria (tricuspid inflow Doppler and tricuspid annular tissue Doppler values measured in the DTGRVIO view) Disagreement of ≥1 grade: 39.5%. Disagreement of 2 grades: 12.3% Observed weighted agreement proportion: 0.74. Expected weighted agreement proportion: 0.63. Linear (Cicchetti Allison) weighted κ=0.3 (95% CI: 0.1 to 0.51; p=0.002), used to evaluate the observed level of agreement beyond chance ASE: American Society of Echocardiography; CI: confidence interval; RVDD: right ventricular diastolic dysfunction; TEE-DTGRVIO: transesophageal echocardiographic deep transgatric right ventricular inflow-outflow view; TTE: transthoracic echocardiography

RVDD grades	RVDD grade (ASE criteria) by TEE (DTGRVIO), n (%)				
RVDD (ASE criteria) by TTE		Normal	Impaired relaxation	Pseudo-normal	Total
	Normal	37 (45.7%)	6 (7.4)	6 (7.4%)	49
	Impaired relaxation	10 (12.3%)	7 (8.6%)	4 (2.5%)	21
	Pseudo-normal	4 (4.9%)	2 (2.5%)	5 (6.2%)	11
	Total	51	15	15	81

TTE vs. TEE (MERVIO) using BSE criteria

Out of 81 paired measurements, disagreement in RVDD grade occurred in nine (11.1%) patients, among which RVDD grade changed from “normal” to “abnormal” from TTE to TEE (MERVIO) in five patients, and from “abnormal” to “normal” in four patients (Table 8). The observed unweighted agreement proportion was 0.889, whereas the expected unweighted agreement proportion was 0.852, yielding an unweighted κ of 0.25 (95% CI: −0.22 to 0.71; p=0.295), indicating fair agreement that was likely to have occurred due to random chance.

**Table 8:** Contingency table assessing agreement between RVDD grading measured by TTE and TEE using the BSE criteria (tricuspid inflow Doppler and tricuspid annular tissue Doppler values measured in the MERVIO view) Disagreement: 11.1%. Observed unweighted agreement proportion: 0.889. Expected unweighted agreement proportion: 0.852. Cohen’s unweighted κ: 0.25 (SE=0.24; 95% CI: –0.22 to 0.71; p=0.295) BSE: British Society of Echocardiography; CI: confidence interval; RVDD: right ventricular diastolic dysfunction; SE: standard error; TEE-MERVIO: transesophageal echocardiographic mid-esophageal right ventricular inflow-outflow view; TTE: transthoracic echocardiography

RVDD grades	RVDD grade (BSE criteria) by TEE (MERVIO), n (%)			
RVDD (BSE criteria) by TTE		Normal	Abnormal	Total
	Normal	70 (86.4%)	5 (6.2%)	75
	Abnormal	4 (4.9%)	2 (2.5%)	6
	Total	74	7	81

TTE vs. TEE (DTGRVIO) using BSE criteria

Disagreement in RVDD grade occurred in 12 (14.8%) of 81 paired measurements, where RVDD grade changed from “normal” to “abnormal” from TTE to TEE (DTGRVIO) in nine patients, and from “abnormal” to “normal” in three patients (Table 9). The observed unweighted agreement proportion was 0.852, whereas the expected unweighted agreement proportion was 0.799, yielding an unweighted κ of 0.260 (95% CI: −0.13 to 0.65; p=0.187), indicating fair agreement that was likely to have occurred due to random chance.

**Table 9:** Contingency table assessing agreement between RVDD grading measured by TTE and TEE using BSE criteria (tricuspid inflow Doppler and tricuspid annular tissue Doppler values measured in the DTGRVIO view) Disagreement: 14.8%. Observed unweighted agreement proportion: 0.852. Expected unweighted agreement proportion: 0.799. Cohen’s unweighted κ: 0.260 (SE=0.2; 95% CI: –0.13 to 0.65; p=0.187). BSE: British Society of Echocardiography; CI: confidence interval; RVDD: right ventricular diastolic dysfunction; SE: standard error; TEE-DTGRVIO: transesophageal echocardiographic deep transgatric right ventricular inflow-outflow view; TTE: transthoracic echocardiography

RVDD grades	RVDD (BSE criteria) by TEE (DTGRVIO), n (%)			
RVDD (BSE criteria) by TTE		Normal	Abnormal	Total
	Normal	66 (81.5%)	9 (11.1%)	75
	Abnormal	3 (3.7%)	3 (3.7%)	6
	Total	69	12	81

**Figure 2:**
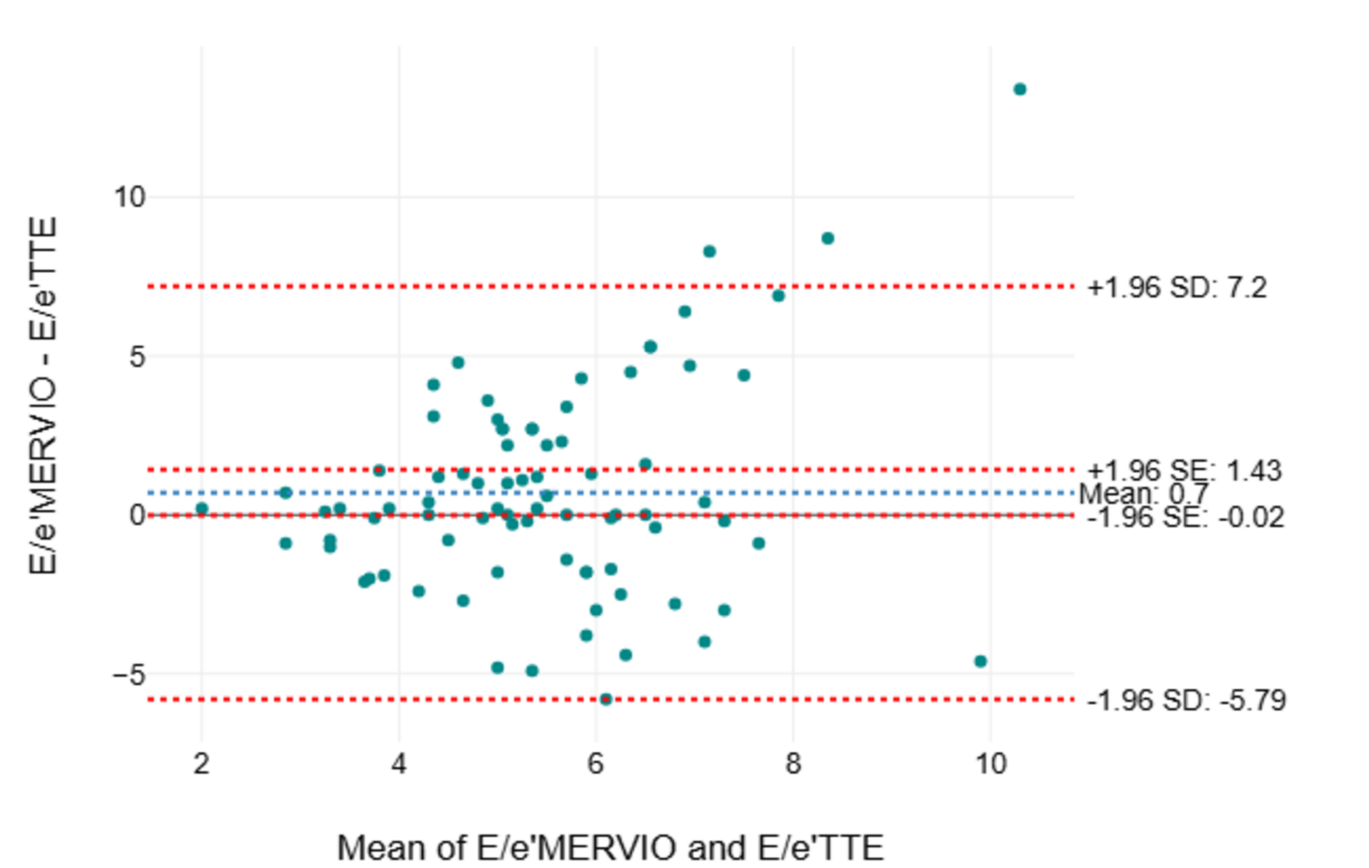
Bland-Altman plot of differences in measured values of E/e’ between TTE (E/e’TTE) and TEE (MERVIO view E/e’MERVIO) Bland-Altman plot of differences in measured values of E/e’ between TTE (E/e’TTE) and TEE (MERVIO view E/e’MERVIO) in the Y-axis against the mean of the values measured in the X-axis with a mean bias of 0.7 (95% CI of bias: −0.02 to 1.43), an upper limit of agreement of 7.2, and a lower limit of agreement of −5.79 CI: confidence interval; MERVIO: mid-esophageal right ventricular inflow-outflow; SD: standard deviation, SE: standard error; TEE: transesophageal echocardiography; TTE: transthoracic echocardiography

**Figure 3:**
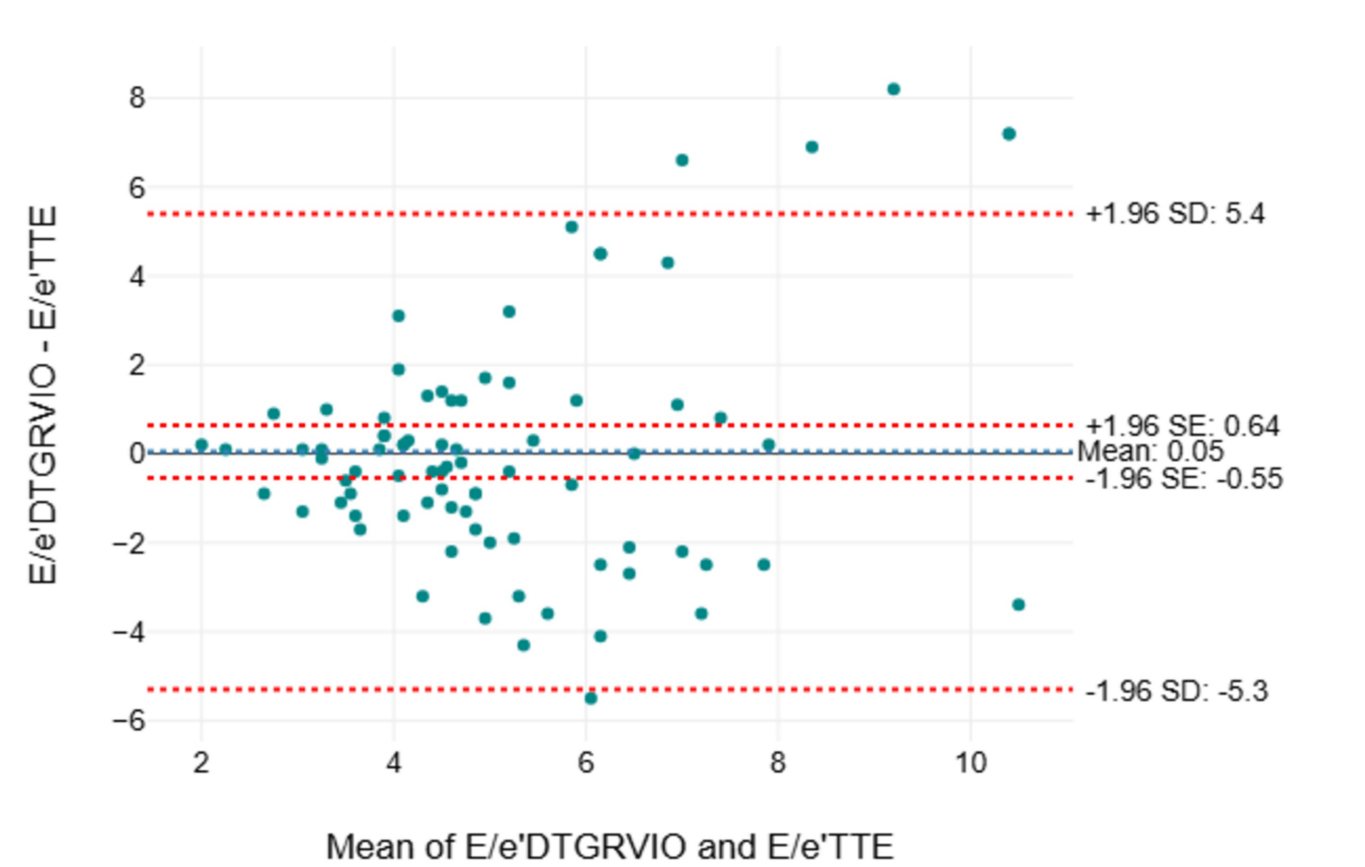
Bland-Altman plot of differences in measured values of E/e’ between TTE (E/e’TTE) and TEE (DTGRVIO view E/e’DTGRVIO) Bland-Altman plot of differences in measured values of E/e’ between TTE (E/e’TTE) and TEE (DTGRVIO view E/e’DTGRVIO) in the y-axis against the mean of the values measured in the x-axis with a mean bias of 0.05 (95% CI of bias: −0.55 to 0.64), an upper limit of agreement of 5.4, and a lower limit of agreement of −5.3. CI: confidence interval; DTGRVIO: deep transgastric right ventricular inflow-outflow; SD: standard deviation, SE: standard error; TEE: transesophageal echocardiography; TTE: transthoracic echocardiography

The Bland-Altman analysis of agreement between individual 2D and Doppler parameters measured by TTE and TEE showed significant negative bias with wide limits of agreement for E velocity, A velocity, e’ velocity, a’ velocity, E wave deceleration time (EDT), and hepatic vein (HV) D wave measured by TEE, whereas E/A ratio, E/e’ ratio, and e’/a’ ratio had small, insignificant bias but with wide limits of agreement (Table 10). The E/e’ ratio also showed proportional bias, with the difference between values measured by TTE and TEE increasing when the mean of TTE- and TEE-measured values exceeded 6 (Figures 2-3).

**Table 10:** Agreement between 2D and Doppler parameters measured by TEE and TTE (tricuspid inflow Doppler and tricuspid annular tissue Doppler measured in both MERVIO and DTGRVIO views) Data presented as bias and limits of agreement EDT: E wave deceleration time; DTGRVIO: deep transgastric right ventricular inflow-outflow view; HV: hepatic vein; IVC: inferior vena cava; IVRT: isovolumic relaxation time; LOA: level of agreement; MERVIO: mid-esophageal right ventricular inflow-outflow view; RA: right atrium; RV: right ventricle; SFfr: systolic filling fraction; TEE: transesophageal echocardiography; TTE: transthoracic echocardiography

Parameter	View	Bias	Upper LOA	Lower LOA
E velocity (cm/s)	MERVIO	−7.6	19.5	−34.6
	DTGRVIO	−11.5	12.9	−35.8
A velocity (cm/s)	MERVIO	−10.9	19.1	−41.0
	DTGRVIO	−12.6	15.3	−40.5
e’ velocity (cm/s)	MERVIO	−2.4	4.1	−8.8
	DTGRVIO	−2.3	3.6	−8.0
a’ velocity (cm/s)	MERVIO	−2.1	6.3	−10.5
	DTGRVIO	−2.9	3.9	−9.7
E/A ratio	MERVIO	0.12	1.1	−0.8
	DTGRVIO	0.04	0.99	−0.91
E/e’ ratio	MERVIO	0.7	7.2	−5.8
	DTGRVIO	0.05	5.4	−5.3
e’/a’ ratio	MERVIO	−0.02	0.9	−0.9
	DTGRVIO	0.04	0.7	−0.6
EDT (ms)	MERVIO	−29	156	−215
	DTGRVIO	−18	177	−212
IVRT (ms)	MERVIO	−2.5	41.7	−46.7
	DTGRVIO	−0.8	34.6	−36.2
RA area (cm^2^)		1.3	7.9	−5.5
Indexed RA area (cm^2^/m^2^)		0.74	4.8	−3.3
RV wall thickness (mm)		−0.2	2.0	−2.4
IVC diameter (cm)		0.1	0.8	−0.6
HV S wave (cm/s)		−7.0	16.9	−30.9
HV D wave (cm/s)		−3.9	8.7	−16.6
HV S/D ratio		−0.1	0.9	−1.1
HV SFfr (%)		−1.4	15.5	−18.2

Inter-rater agreement for RVDD grade

The observed weighted agreement proportion between the two independent evaluators regarding RVDD grade measured by TTE according to the ASE criteria was 0.98, and the expected weighted agreement proportion was 0.64. The linear weighted κ (0.93) indicated that there was almost perfect agreement between the two evaluators [standard error (SE)=0.03; 95% CI: 0.87 to 1; p=0.000). The observed agreement proportion between the two independent evaluators regarding RVDD grade measured by TTE according to the BSE criteria was 0.98, and the expected agreement proportion was 0.86. The unweighted κ (0.82) showed that there was almost perfect agreement between the two evaluators (SE=0.13; 95% CI: 0.57 to 1.07; p=0.000).

The observed weighted agreement proportion between the two independent evaluators regarding RVDD grade measured by TEE (MERVIO) according to the ASE criteria was 0.94, and the expected weighted agreement proportion was 0.65. The linear weighted κ (0.82) showed that there was almost perfect agreement between the two evaluators (SE=0.05; 95% CI: 0.72 to 0.93; p=0.000). The observed agreement proportion between the two independent evaluators regarding RVDD grade measured by TEE (MERVIO) according to the BSE criteria was 0.95, and the expected agreement proportion was 0.84. The unweighted κ (0.69) showed that there was substantial agreement between the two evaluators (SE=0.15; 95% CI: 0.39 to 0.99; p=0.000).

The observed weighted agreement proportion between the two independent evaluators regarding RVDD grade measured by TEE (DTGRVIO) according to the ASE criteria was 0.94, and the expected weighted agreement proportion was 0.62. The linear weighted κ showed that there was almost perfect agreement between the two evaluators (SE=0.05; 95% CI: 0.76 to 0.94; p=0.000). The observed agreement proportion between the two independent evaluators regarding RVDD grade measured by TEE (DTGRVIO) according to the BSE criteria was 0.95, and the expected agreement proportion was 0.76. The unweighted κ (0.79) showed that there was substantial agreement between the two evaluators (SE=0.1; 95% CI: 0.59 to 0.99; p=0.000).

Inter-rater reliability between both the independent evaluators for all the 2D and Doppler parameters measured by both TTE and TEE (tricuspid inflow Doppler and tricuspid annular tissue Doppler parameters in both MERVIO and DTGRVIO views), as evaluated by intra-class correlation coefficient, showed almost perfect agreement (ICC >0.8) for all the parameters.

(Supplemental Figures 4-11 in the Appendices depict representative images of the RVDD parameters measured by TTE and TEE).

## Discussion

Our study found only a fair agreement between RVDD grades assessed by TTE and TEE using the ASE criteria, with the tricuspid inflow Doppler and tricuspid annular tissue Doppler velocities measured in the DTGRVIO view in TEE. There was no significant agreement beyond random chance between RVDD grades assessed by TTE and TEE using the BSE criteria (with the tricuspid inflow Doppler and tricuspid annular tissue Doppler velocities measured in both MERVIO and DTGRVIO views in TEE) as well as using the ASE criteria, with the tricuspid inflow Doppler and tricuspid annular tissue Doppler velocities measured in the MERVIO view in TEE. There were wide limits of agreement between TTE and TEE measurements of individual 2D, Doppler, and tissue Doppler parameters required for the assessment of the RVDD grades. However, there was almost perfect agreement between the two independent observers in the assessment of RVDD grades for both the ASE and BSE criteria using measurements obtained by both TTE and TEE. There was also excellent agreement between the observers for the majority of the measured 2D, Doppler, and tissue Doppler parameters using both TTE and TEE.

The secondary objective of this study was to determine the TEE view with the best agreement with TTE for the assessment of RVDD grade. It was observed that the best agreement happened between TTE and TEE regarding RVDD grade when tricuspid inflow Doppler and tricuspid annular tissue Doppler parameters measured in the DTGRVIO view were used. To the best of our knowledge, this is the first study to investigate the agreement between TTE and TEE regarding RVDD grades, which we evaluated using both the relatively brief ASE criteria and also the more extensive BSE criteria. Several studies in the literature have examined agreement between TTE and TEE in grading LV diastolic dysfunction, as well as in assessing RV systolic function.

McIlroy et al. [7] examined the agreement between LV diastolic dysfunction grades measured by preoperative TTE (TTE awake) and intraoperative TEE (TEE anesth) in 98 patients scheduled for cardiac surgery by measuring key Doppler parameters at different time points: before anesthesia induction (TTE awake), after induction (TTE anesth), and intraoperatively (TEE anesth). The findings indicated poor agreement of LV diastolic dysfunction grades measured by TTE preinduction and TEE post-induction, with discrepancies of ≥1 grade in 43 (54%) and ≥2 grades in eight (10%) out of the 79 patients. In addition, when comparing TTE anesth with TEE anesth, disagreement by ≥1 grade was observed in 50 patients (60%) and by ≥2 grades in 10 patients (12%) out of 83 paired measurements. The observed weighted agreement proportion between TTE anesth and TEE anesth was 0.76, whereas the expected weighted agreement proportion was 0.67, resulting in a weighted κ of 0.26 (95% CI: 0.12-0.41) for the observed level of agreement beyond chance. Therefore, the results reflected poor agreement between the LV diastolic dysfunction grades measured by TTE and TEE under similar anesthetic influence.

Several studies have investigated the agreement of different echocardiographic parameters used in assessing LV diastolic function between TTE and TEE. Aksakal et al. [8] reported a mean difference in e’average of +2 cm/s when measured by TEE compared to TEE, with 95% limits of agreement of −4 to +6 cm/s, concluding that TTE and TEE could be used interchangeably for evaluating diastolic function. In contrast, Nilsson et al. [9] reported a reduction in e’lat and e’sept of 37% (6.32 ±1.73 cm/s vs. 10.01 ±3.01 cm/s) and 17% (6.35 ±1.56 cm/s vs. 7.67 ±2.61 cm/s), respectively, when measured by TEE as compared to TTE. Based on these results, the authors suggested that TTE reference values should not be used interchangeably with measurements made by TEE.

Mauermann et al. [10] reported a slight positive difference for e’average when measured by TTE anesth compared to TEE anesth, with 95% limits of agreement between −2.9 to 1.8 cm/s, and determined that the differences observed were not significant clinically. However, none of these studies indicated the impact of discrepancies in individual Doppler measurements on the assessment of diastolic dysfunction, as done by McIlroy et al. [7]. Roberts et al. [6] evaluated the agreement between TEE and TTE in measuring RV function using standard 2D and Doppler methods among adult patients undergoing elective cardiac surgery. Key measurements included tricuspid annular plane systolic excursion, fractional area change, right-sided index of myocardial performance, and tricuspid annular systolic velocity. The agreement of parameters was assessed using the concordance correlation coefficient and paired t-tests. The results indicated a poor correlation between TEE and TTE measurements, suggesting that TEE data should be cautiously interpreted when extrapolating from TTE-validated data.

The disagreements observed in our study were mainly by 1 grade according to the ASE criteria. The minimal detectable change for assessing diastolic dysfunction has not yet been established, especially for grading RVDD, and the minutest of differences in parameters may alter the assigned grade of dysfunction, possibly by more than 1 grade if a threshold is crossed. Compared to the ASE criteria, disagreement occurred between TTE and TEE in a smaller proportion of cases when RVDD was graded according to the BSE criteria (11.1% using tricuspid inflow Doppler and tricuspid annular Doppler parameters measured in MERVIO view; 14.8% using tricuspid inflow Doppler and tricuspid annular Doppler parameters measured in DTGRVIO view). Instead of classifying RVDD into more distinct grades such as “impaired relaxation,” “pseudo-normal,” and “restrictive,” similar to that done for grading LV diastolic dysfunction, the BSE criteria recommend that users simply differentiate between “normal” and “abnormal” RV diastolic function by using an extensive number of parameters, comprising both 2D- and Doppler-derived parameters [5].

Compared to the ASE criteria, a higher proportion of patients were assigned a “normal” grade by the BSE criteria in our study when using both TTE- and TEE-derived parameters (92.6%, 91.4%, and 85.2% compared with 60.5%, 63%, and 45.7% by TTE, TEE-MERVIO, and TEE DTGRVIO, respectively). In the absence of similar data comparing the ASE and BSE criteria for grading RVDD, the significance of this finding cannot be properly explained. However, the difference in grading diastolic dysfunction by two different criteria is not an unexpected finding. In a study by Luke et al. [11] on the agreement between ASE and BSE criteria for grading LV diastolic dysfunction, significant disagreement was found between the gradings by the two criteria, with the latter classifying a significantly higher proportion of patients as having abnormal and indeterminate diastolic function compared to the former. In our study, the incorporation of a greater number of parameters by the BSE criteria, thereby making the grading system more extensive, for classifying RVDD in comparison to the ASE criteria, is the most plausible explanation for this discrepancy between grading by ASE and BSE criteria.

Various factors may explain the poor agreement between TTE and TEE for grading diastolic dysfunction, with the foremost reason being random variability between the key Doppler variables [7]. Regarding Doppler parameters, beat-to-beat variability has the potential to impact algorithm-guided grading, which has been emphasized in previous studies [12]. Therefore, it is recommended to average the parameter values over numerous successive cardiac cycles. A study comprising 50 patients undergoing repeated TTE examination on the same day exhibited high coefficients of variation for both e’lat (26%) and E/e’lat (36%) [13]. Nevertheless, disagreement in grading by the ASE/EACVI algorithm occurred in only 13 out of 50 patients, and never by more than 1 grade. Moreover, all the measurements in our study were done at deliberate end-expiration apnea, with the Doppler parameters averaged over five cardiac cycles, thereby reducing the possibility of random variability in key Doppler variables affecting the results of our study.

The second most probable cause of the poor agreement between TTE and TEE is systematic measurement error due to the use of diverse imaging modalities for measuring the same parameters under different physiological conditions [7]. However, the hemodynamic, anesthetic, and ventilatory parameters were comparable during TTE and TEE, thereby ruling out the possibility of differences in physiological conditions affecting the diastolic dysfunction gradings. Unlike the assessment of LV diastolic dysfunction by TEE, where both the blood flow through mitral valve as well as the mitral annular motions can be perfectly aligned to the Doppler and the tissue Doppler beams, respectively, due to location of the TEE transducer being just behind the left atrium in the mid-esophagus, the assessment of RVDD by TEE is not straightforward due to the anterior location of the RV within the thoracic cavity. RV being far from the TEE transducer, as well as the complex three-dimensional crescentic shape of the RV, makes the blood flow through the TV, as well as the tricuspid annular motion, difficult to align with the Doppler beam and the tissue Doppler beam, respectively [14].

In TTE, the position of the transducer can be varied over the chest wall to obtain the best alignment of the Doppler beam with the tricuspid inflow, as well as the tricuspid annular motion. In contrast, the position of the TEE transducer is relatively fixed within the esophagus or the stomach, leaving little room for manipulation to achieve optimal alignment of the Doppler beam with the RV inflow and the motion of the tricuspid annulus. Extreme changes in the scan angle in the ranges of 60-70° and 110-120° are required to obtain optimal alignment. Furthermore, the tricuspid annulus has a complex, non-planar structure that is elliptical as well as saddle-shaped, with two superiorly oriented high points and two inferiorly oriented low points [15]. The motion of the tricuspid annulus is also asymmetrical [6]. Due to the complexity of its shape, the RV appears to be triangular in some echocardiographic views, whereas it is crescentic in some other views.

The combination of all of these factors contributes to the poor agreement of the Doppler-measured values between TTE and different views of TEE, as the angle of interrogation of blood flow through the TV, as well as of the motion of the tricuspid annulus, vary significantly between different views. For example, what seems to be a good alignment of the RV inflow and/or the tricuspid annular motion in a particular TEE view may not be aligned with the true direction of blood flow or annular motion [6]. Moreover, the portion of the RV inflow and the RV wall interrogated also differs significantly between TTE and different views of echocardiography. The wide limits of agreement between 2D and Doppler parameters measured by TTE and TEE in our study support this explanation. Although the net effect of differences in key Doppler variables measured by TEE compared by TTE on grading is unpredictable, the combined effect of the wide limits of agreement of all key parameters, including a proportional bias of E/e’ above a value of 6 (i.e., the cut-off value in both the ASE and BSE criteria between normal and abnormal) is expected to cross grading thresholds.

Another reason for the discrepancy could be that TEE effectively identifies genuine changes in grades of diastolic dysfunction amidst the two time points. Intraoperative TEE allows for repetitive, real-time assessment of diastolic function during surgery [7]. Previous studies have investigated the impact of continuous intraoperative assessment of diastolic dysfunction using TEE, although the effectiveness of this approach is uncertain [16]. However, the gap between TTE and TEE examinations in our study was only 10 minutes before giving any surgical stimulus, in addition to hemodynamic, anesthetic, and ventilatory parameters remaining comparable between the time points, thus lowering the possibility of TEE detecting a real change in diastolic function to almost negligible. Additionally, identifying true changes accurately in diastolic function is difficult without an easily accessible gold-standard diagnostic aid for evaluating diastolic dysfunction.

The major strength of our study is that the TTE and TEE measurements were taken within a short period, with similar hemodynamic, anesthetic, and ventilatory conditions, and measurements were done during deliberate end expiration apnea, with values averaged over five cardiac cycles, thereby reducing potential confounders that could affect our results. There was no significant inter- or intra-rater variability in the measured 2D and Doppler parameters, or the grading of RVDD by both TTE and TEE, thereby ruling out the possibility of inter- or intra-individual variation in grading confounding our results.

Study limitations

Our study has a few limitations. We did not perform test-retest variability analysis for the parameters obtained for our patients, leading to the possibility of subjective variation while acquiring the required imaging views. Secondly, the study did not look into, nor was it adequately powered to assess, the prognostic impact of the disagreement between the two modalities regarding RVDD grades. Whether patients whose RVDD grade detected by TEE was different than that detected by TTE had any significant perioperative hemodynamic perturbation, potentially attributable to changes in RV diastolic function, was also not considered. None of the patients was graded as having “restrictive” RVDD in our study in terms of either ASE or BSE criteria as measured by both TTE and TEE. Our study population comprised mainly patients with CABG or valvular heart disease, in whom restrictive physiology is uncommon. Restrictive RV diastolic function has been mainly reported in tetralogy of Fallot patients after intra-cardiac repair [17], who were not included in this study. However, the absence of “restrictive” grading in the whole study cohort did not affect the overall outcome of our study.

## Conclusions

Our study found that there was, at most, only a fair agreement between TTE and TEE in terms of assessing RVDD grades based on ASE and BSE criteria and that inter-rater variability was not significant. There were also wide limits of agreement of the measured echocardiographic parameters between TTE and TEE. The best agreement (weighted κ=0.3) was found when RVDD was graded using the tricuspid inflow Doppler and tricuspid annular tissue Doppler parameters measured in the DTGRVIO view by TEE, using the ASE criteria. Further studies are required to develop an algorithm to grade RVDD through TEE and to analyze the prognostic efficacy of TTE and TEE for clinically relevant adverse outcomes related to RVDD.

## Figures and Tables

**Figure 4:**
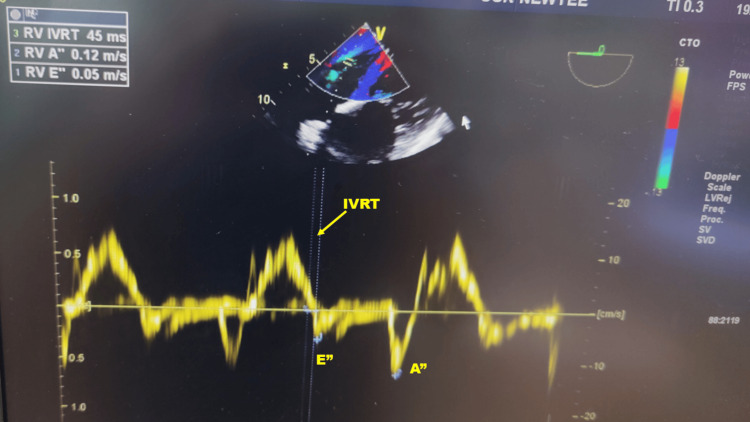
Tricuspid annular TDI by TEE in DTGRVIO view A": a' velocity; E": e' velocity; DTGRVIO: deep transgastric right ventricular inflow-outflow; IVRT: isovolumic relaxation time; RV: right ventricle; TDI: tissue Doppler imaging; TEE: transesophageal echocardiography

**Figure 5:**
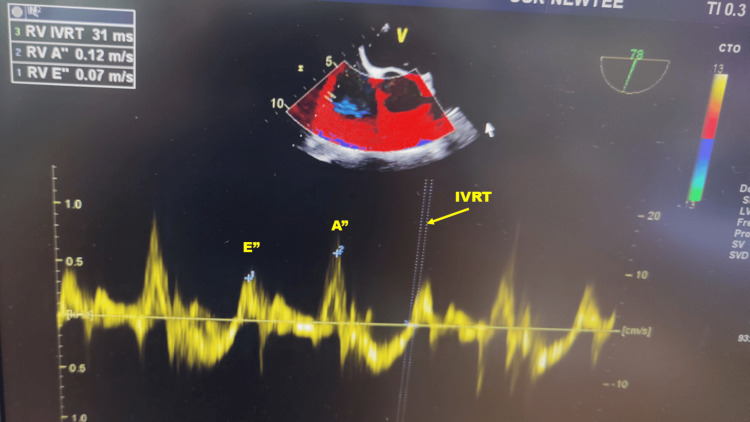
Tricuspid annular TDI by TEE in MERVIO view A": a' velocity; E": e' velocity; IVRT: isovolumic relaxation time; MERVIO: mid-esophageal right ventricular inflow-outflow; RV: right ventricle; TDI: tissue Doppler imaging; TEE: transesophageal echocardiography

**Figure 6:**
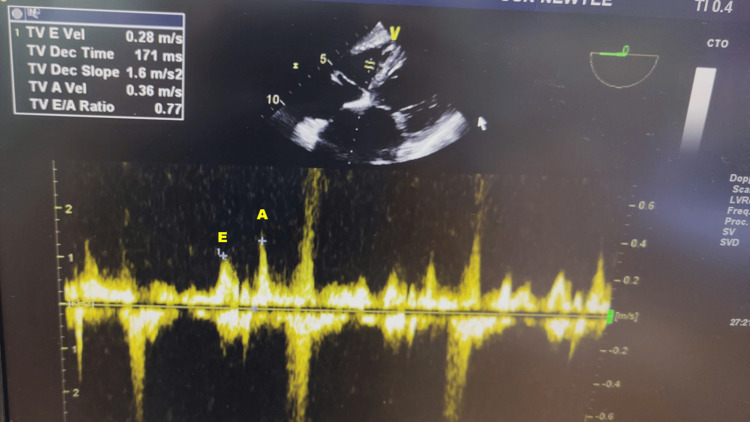
Tricuspid inflow Doppler by TEE in DTGRVIO view A vel: A wave velocity; Dec: deceleration; DTGRVIO: deep transgastric right ventricular inflow-outflow; E vel: E wave velocity; TEE: transesophageal echocardiography; TV: tricuspid valve

**Figure 7:**
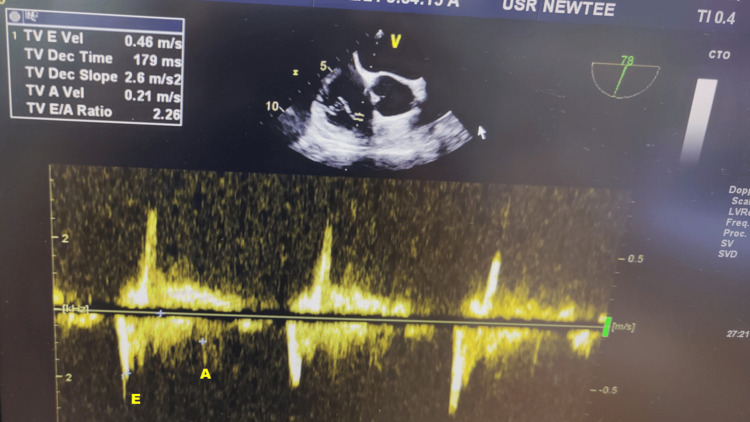
Tricuspid inflow Doppler by TEE in MERVIO view A vel: A wave velocity; Dec: deceleration; E vel: E wave velocity; MERVIO: mid-esophageal right ventricular inflow-outflow; TEE: transesophageal echocardiography; TV: tricuspid valve

**Figure 8:**
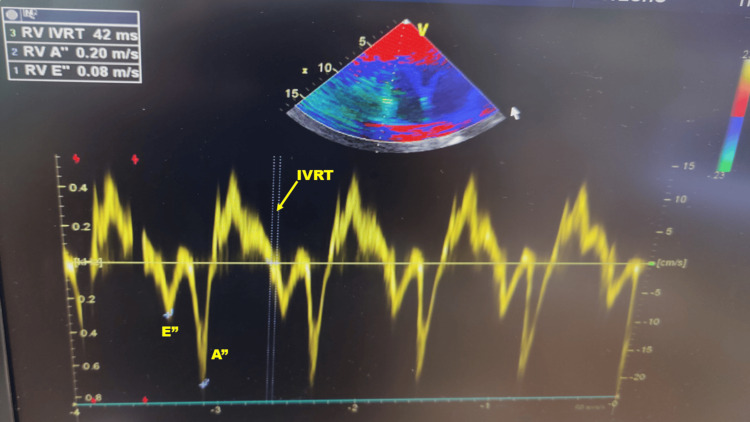
Tricuspid annular TDI by TTE A": a' velocity; E": e' velocity; IVRT: isovolumic relaxation time; RV: right ventricle; TDI: tissue Doppler imaging; TTE: transthoracic echocardiography

**Figure 9:**
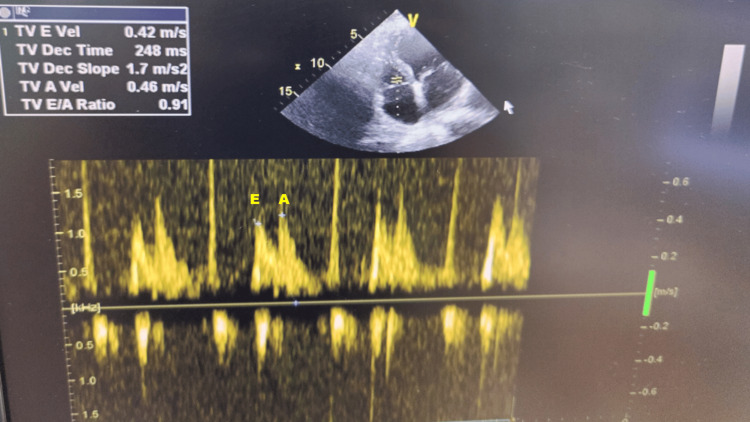
Tricuspid inflow Doppler by TTE A vel: A wave velocity; Dec: deceleration; E vel: E wave velocity; TTE: transthoracic echocardiography; TV: tricuspid valve

**Figure 10:**
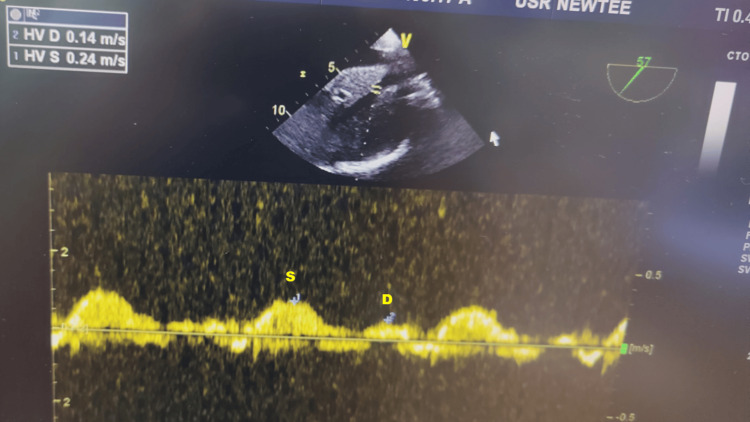
Hepatic venous Doppler by TEE D: diastolic wave; HV: hepatic vein; S: systolic wave; TEE: transesophageal echocardiography

**Figure 11:**
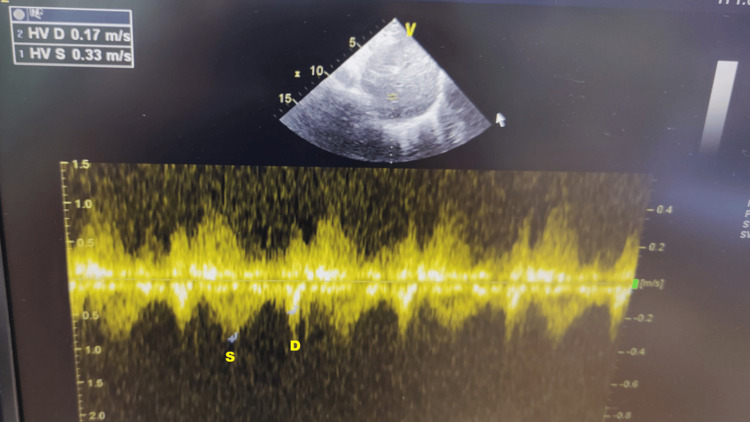
Hepatic venous Doppler by TTE D: diastolic wave; HV: hepatic vein; S: systolic wave; TTE: transthoracic echocardiography
